# 12382 Circadian Disruption in Pancreatic Cancer Carcinogenesis

**DOI:** 10.1017/cts.2021.421

**Published:** 2021-03-30

**Authors:** Patrick B. Schwartz, Morgan T. Walcheck, Kristina A. Matkowskyj, Christopher A. Bradfield, Sean M. Ronnekleiv-Kelly

**Affiliations:** 1 Department of Surgery, University of Wisconsin School of Medicine and Public Health; 2 Department of Pathology and Laboratory Medicine, University of Wisconsin School of Medicine and Public Health; 3 Department of Oncology, University of Wisconsin School of Medicine and Public Health; 4 Department of Surgery, Division of Surgical Oncology, University of Wisconsin School of Medicine and Public Health

## Abstract

ABSTRACT IMPACT: Circadian disruption is known to cause significant human pathology but has not been evaluated in pancreas cancer carcinogenesis; through understanding how disruption of circadian rhythms can lead to pancreas cancer development and spread, preventive and therapeutic strategies can be devised. OBJECTIVES/GOALS: Pancreatic ductal adenocarcinoma (PDAC) is a lethal cancer due to early spread and poor response to therapy. Identifying factors driving PDAC growth could lead to new therapeutic strategies. Thus, we evaluated the extent to which circadian rhythm disruption, a factor strongly associated with cancer formation, contributes to PDAC pathogenesis. METHODS/STUDY POPULATION: To achieve the objective, we evaluated mice with pancreas lineage Kras-mutation (KC mice), which are predisposed to develop the full spectrum of pancreas cancer precursor lesions (pancreatic intra-epithelial neoplasia or PANIN-1, 2, 3) and PDAC. We subjected KC mice to a light-dark phase shift protocol known to induce circadian disruption (KCCD, n = 18), and another group to standard lighting conditions (KCNC, n = 31), with equal numbers of males and females in each group. The mice were allowed access to food and water ad libitum until sacrifice at age 9 months. Histopathologic evaluation of the pancreas was then performed to assess for pancreatic inflammation, pancreatic precursor lesions (PANIN) and PDAC. Fisher’s Exact Test was used to evaluate differences in incidence. RESULTS/ANTICIPATED RESULTS: As expected, both groups of mice demonstrated 100% incidence of chronic pancreatitis and PANIN-1 (low-grade precursor lesion) at age 9 months. This is consistent with the KC phenotype. However, the KCCD mice demonstrated a significant increase in acute pancreatic inflammation (61.1% vs 19.4%, p = 0.005) compared to KCNC mice. Furthermore, intermediate grade precursor lesions (PANIN-2) were also significantly increase in the KCCD mice (38.9% vs 6.5%, p = 0.006). Incidence of high-grade precursor lesions (PANIN-3, or carcinoma in situ: 22.2% vs 9.7%) and PDAC (27% vs 19%) were also increased, but these were not statistically significant. These results are notable given the established progression from higher grade premalignant PANIN lesions (PANIN-2, PANIN-3) to PDAC. DISCUSSION/SIGNIFICANCE OF FINDINGS: Insight into how circadian disruption leads to increased PANIN-2 formation and increase in acute inflammation may be advantageous for understanding circadian disruption in PDAC carcinogenesis. The circadian clock is present in immune cells and disruption can induce immune dysregulation. This mechanism will be evaluated in follow up studies.

